# Structural mechanism of Fab domain dissociation as a measure of interface stability

**DOI:** 10.1007/s10822-023-00501-9

**Published:** 2023-03-15

**Authors:** Nancy D. Pomarici, Franz Waibl, Patrick K. Quoika, Alexander Bujotzek, Guy Georges, Monica L. Fernández-Quintero, Klaus R. Liedl

**Affiliations:** 1 Institute of General, Inorganic and Theoretical Chemistry, and Center for Molecular Biosciences Innsbruck (CMBI), University of Innsbruck, Innrain 80-82, 6020 Innsbruck, Austria; 2 Center for Protein Assemblies (CPA), Physics Department, Chair of Theoretical Biophysics, Technical University of Munich, Ernst-Otto-Fischer-Str. 8, 85748 Garching, Germany; 3 Roche Pharma Research and Early Development, Large Molecule Research, Roche Innovation Center Munich, Nonnenwald 2, 82377 Penzberg, Germany

**Keywords:** Fab stability, Dissociation mechanism, Interface stability, Antibody design, Molecular dynamics

## Abstract

Therapeutic antibodies should not only recognize antigens specifically, but also need to be free from developability issues, such as poor stability. Thus, the mechanistic understanding and characterization of stability are critical determinants for rational antibody design. In this study, we use molecular dynamics simulations to investigate the melting process of 16 antigen binding fragments (Fabs). We describe the Fab dissociation mechanisms, showing a separation in the V_H_–V_L_ and in the C_H_1–C_L_ domains. We found that the depths of the minima in the free energy curve, corresponding to the bound states, correlate with the experimentally determined melting temperatures. Additionally, we provide a detailed structural description of the dissociation mechanism and identify key interactions in the CDR loops and in the C_H_1–C_L_ interface that contribute to stabilization. The dissociation of the V_H_–V_L_ or C_H_1–C_L_ domains can be represented by conformational changes in the bend angles between the domains. Our findings elucidate the melting process of antigen binding fragments and highlight critical residues in both the variable and constant domains, which are also strongly germline dependent. Thus, our proposed mechanisms have broad implications in the development and design of new and more stable antigen binding fragments.

**Supplementary Information:**

The online version contains supplementary material available at 10.1007/s10822-023-00501-9.

## Introduction

Thanks to their specificity and their ability to target diverse molecules, monoclonal antibodies (mAbs) have emerged as an important class of biopharmaceuticals [1–3]. However, many antibodies exhibit several issues like self-association, inadequate pharmacokinetics, immunogenic response that may raise production costs and limit their functionality [3–5]. In particular, antibodies’ instability may cause important clinical consequences, like increased aggregation propensity, fast clearance or immunogenic response [6–8].

In the process of antibody design, mutations are accumulated in the binding fragment to optimize the antibodies and improve their affinity. However, this may result in an unwanted destabilization of the structure [9–11]. Additional modifications are thus required to guarantee thermodynamic stability [12]. Therefore, antibodies’ structure needs to be characterized in detail, in order to increase their stability and to help molecular engineering in the production of suitable therapeutics.

The ability of antibodies to specifically recognize their antigens is determined by the antigen binding fragment (Fab). The Fab consists of a heavy and a light chain (LC) and it can be subdivided in a variable (Fv) and a constant region (C_H_1–C_L_). The antigen-binding site is located at the top of the Fv region, and it consists of six loops, known as the complementarity determining regions (CDRs). Thanks to their hypervariable sequences and to the numerous conformations that they can adopt in solution, the CDRs give the antibodies the ability to bind a wide repertoire of antigens [13, 14]. Many of the affinity-enhancing mutations happen in the variable V_H_–V_L_ interface [4]. Changes in this area improve the complementarity of the binding site towards the antigen, modifying the loop conformations or rotating the V_H_–V_L_ orientation [15–17]. Mutations in the paratope to achieve higher affinity are reflected in a consequent loss of flexibility of the loops [10, 16, 18]. Specific point mutations in the CDR-H3 and CDR-L3 loops also contribute to achieve a high bispecific IgG yield and avoid HC–LC mispairing [19, 20]. The loop region not only determines the ideal folding for the binding, but it contributes, together with the protein core, to stabilize the structure [21]. Therefore, optimization of both the CDR region and the framework can substantially enhance biophysical properties like stability [22, 23].

The two Fab constant domains, C_H_1–C_L_, play an important role in stabilizing the Fab and in the binding process. In fact, not only a mutual stabilization occurs across the V_H_–V_L_ and C_H_1–C_L_ interfaces, but also the C_H_1–C_L_ domains can improve the antigen–antibody complementarity [24, 25]. Engineering the interface of antibodies is currently a well-established technique to obtain novel and better performing therapeutics, as bispecific antibodies [26, 27]. The first engineering attempts led to a successful heterodimerization of the two heavy chains (HCs), by modifying few residues in the third constant domain, the C_H_3–C_H_3, using the Knob-into-Hole (KiH) technology or charge inversions [28–30]. More recently, the introduction of KiH mutations and charge inversions in the Fab interfaces or applying the domain crossover addressed the challenge of the correct LC–HC pairing, to obtain the right antigen binding fragment [31]. Consequently, the study of the C_H_1–C_L_ interface is highly relevant to help the development of novel antibodies formats.

We studied the mechanism of dissociation for a dataset composed by 16 crystal structures in which four HCs were paired with four κ LCs [32], characterizing the key determinants for antibody stability. We reveal the role of the CDR loops in the dissociation process and describe possible mechanisms of dissociation in the Fv and in the C_H_1–C_L_, pointing out the contacts that are relevant to keep the domains together. The detailed analyses of the instability of the Fab structures and the mechanism of the consequent domains’ dissociation provide an important understanding for modifications and improvements in engineering therapeutic antibodies.

## Methods

### Dataset

Four HC germlines, IGHV1-69 (H1-69), IGHV3-23 (H3-23), IGHV5-51 (H5-51) and IGHV3-53 (H5-53) and four LC germlines (all κ), IGKV1-39 (L1-39), IGKV3-11 (L3-11), IGKV3-20 (L3-20) and IGKV4-1 (L4-1) have been previously combined and crystalized by Teplyakov et al. [32]. These genes have been selected because they are frequently used [33]. The resultant 16 Fab structures differ in their sequences and structures, except for the CDR-H3 loop sequence that is grafted identically to the four HC germlines. The dataset is provided by Teplyakov et al. with experimental information, e.g., crystal structures and melting temperatures [32].

### Structure preparation

**Fig. 1:**
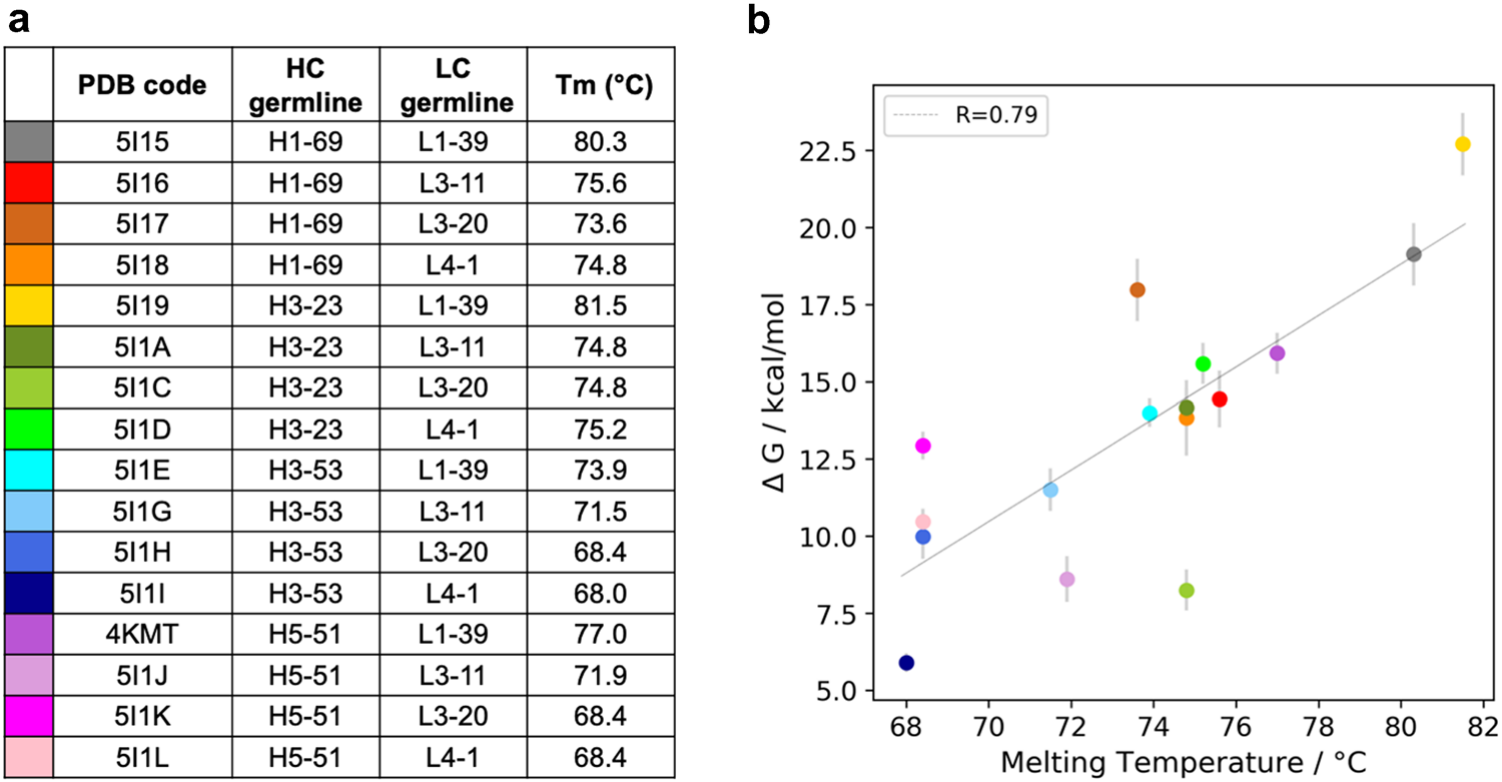
Correlation of the energy depth with the experimentally determined melting temperatures for 16 antigen binding fragments.** a** Table including the PDB codes of the 16 systems (dataset from Teplyakov et al. [30]), the heavy and light chain germline type that they comprise, and their experimentally measured melting temperatures. **b** Correlation between the energy depth of the bound Fab and its melting temperature. Each system is color coded according to **a**. The errors are represented by grey lines

Crystal structures of the 16 antigen binding fragments are available in the Protein Data Bank (PDB) [34] with the PDB codes 5I15, 5I16, 5I17, 5I18, 5I19, 5I1A, 5I1C, 5I1D, 5I1E, 5I1G, 5I1H, 5I1I, 5I1J, 5I1K, 5I1L, 4KMT. Experimental structural information was available for all considered systems. Figure 1a shows the 16 possible HC–LC pairings and the melting temperatures of the resultant Fab structures. The starting structures for simulations were prepared in MOE (Molecular Operating Environment, Chemical Computing Group, version 2018.01) using the Protonate3D tool [35, 36]. We added the missing side chains and modelled the missing loops. We did not include the cysteines forming a disulfide bond at the C-terminal end of the Fab structures, to avoid an additional stabilization of the C_H_1–C_L_ domains. The annotation of the CDR loops follows the Kabat numbering scheme [37]. The germlines differ in loop lengths, therefore the numeration of the same loop in different germlines can differ.

### Molecular dynamics simulations

We solvated the crystal structures in cubic water boxes of TIP3P water molecules with a minimum wall distance of 10 Å to the protein, using tleap tool of the AmberTools19 package [38–40]. Parameters of AMBER force field 14SB were used [41]. Each system was equilibrated using a multistep equilibration protocol [42].

After the equilibration, we performed 1 µs of conventional Molecular Dynamics (cMD) simulations in the NpT ensemble to characterize the bound state of the Fabs. The simulations were performed using CUDA implementation of the particle mesh Ewald method [43]. All bond lengths involving hydrogen atoms were restrained using the SHAKE algorithm, with a time step of 2.0 fs [44]. We used the Berendsen algorithm to set the system to atmospheric pressure (1 bar) [45] and the Langevin thermostat to maintain the temperature of 300 K during the simulations [46].

### Umbrella sampling simulations

The crystal structures were solvated in cubic water boxes of TIP3P water molecules with a minimum wall distance of 25 Å to the protein and then equilibrated, using the setup described above. A bigger box was used to ensure that the protein does not interact with its periodic images during the dissociation of the domains. To induce the dissociation between the domains, Umbrella Sampling (US) [47] simulations were performed in GROMACS [48–51]. As collective variable (CV), we chose the distance between the center of mass (COM) of the LC and HC. We performed pulling simulations on the equilibrated structures, to generate starting structures for US simulations [52]. The pulling took place along the y and z axis over 500 ps, using a spring constant of 1000 kJ mol^−1^ nm^−2^ and a pull rate of 0.005 nm ps^−1^. An asymmetric distribution of sampling windows was used: the umbrella windows extended between a COM distance of 1.7 and 4 nm with a step size of 0.1 nm between 1.7 and 2 nm, a step size of 0.05 nm between 2 and 2.5 nm, and again a step size of 0.1 nm between 2.5 and 4 nm. Such spacing allowed a more detailed sampling of the region in which the dissociation between the domains happens. After a brief NPT equilibration, each window was run for 50 ns at a constant temperature of 310 K, using the same spring constant as in the pulling simulation. The first 5 ns of each umbrella simulation were excluded from the analysis to avoid conformational states that are not fully equilibrated. The convergence of the US runs is assessed via trajectory splitting (SI Fig. 1a).

The multistate Bennett acceptance ratio estimator (MBAR) implemented in pyMBAR has been used to reweight the US trajectories [53]. This allows the estimation of the potential of mean force (PMF) and a direct way to calculate errors. The reweighted PMF curves for each system are plotted in SI Fig. 1b.

Finally, we concatenated the single US runs, in order of increasing CV, obtaining one single simulation for each system.

### Transition from bound state to encounter complex

We identified three descriptors that fully characterize the undissociated state of the system: the fraction of native contacts (q) with respect to the starting structure and the distance between the COM of the two variable domains (d_1_) and of the two constant domains (d_2_). Therefore, these descriptors were calculated for the cMD simulations, to describe the bound state, and US trajectories of each system using cpptraj [39]. The descriptors q, d_1_ and d_2_ show a bell-shaped distribution during the cMDs. To be able to describe the dissociation process, we defined a state function, which is used to classify any structure in our simulation based on the above-mentioned descriptors. To this end, we fitted a decreasing sigmoidal function on the right side of the bell-shaped distribution of d_1_ and d_2_, since the distance between the domains increases while the domains separate. An increasing one, instead, can be fitted on the left side of the bell-shaped distribution of q, since the fraction of native contacts decreases during dissociation (SI Fig. 2a). The parameters (slope and turning point) obtained by the fitting procedure are then used to project the sigmoidal functions on the values of d_1_, d_2_ and q sampled during the US simulations (SI Fig. 2b). To this end, we used a logistic function:

$$\frac{1}{1+{e}^{-s*(x-x0)}},$$

where *s* represents the slope of the curve and *x*0 the turning point.

At this point, we have three logistic functions, covering a [0, 1] y-range, over the range of values sampled in the US for each descriptor. The multiplication of the three sigmoids results in the state function. State 1 corresponds to the bound state, and it smoothly approaches 0, when the dissociation starts. We call the state corresponding to 0 ‘encounter complex’, as it is not bound anymore but not yet completely unbound. The state definition can be applied to identify in each US simulation the point in time in which the whole Fab structure transits from a bound state to an encounter complex (SI Fig. 2c).

### Transition from encounter complex to unbound state

We used the fraction of native contacts and SASA to define the transition from the encounter complex to the unbound state. These descriptors can properly describe the process of interest, and in addition, they reach a plateau when the two domains are completely separated. A sigmoidal function can be fitted on the timeseries of the two descriptors during the US simulations (SI Fig. 3a). The two curves are then normalized and multiplied. This results in a state function, where 1 represents the structure in the bound state and in the process of dissociation and 0 corresponds to the completely unbound structure (SI Fig. 3b). The time of transition from the encounter complex to the unbound state corresponds to the frame in the trajectory where the curve approaches 0.

### Analysis of interface contacts

The interdomain atomic interactions have been computed using the GetContacts software [54]. This software allows to visualize the evolution of the interdomain contacts during the simulation, specifying the type. The so-called flareplots allow a schematic visualization of the development of the contacts over the simulations. An exemplary representation of flareplots is illustrated in SI Fig. 4b. We calculated the overall number of contacts, which include hydrogen bonds (backbone/backbone, side chain/backbone and side chain/side chain), salt bridges, pi-cation, pi-stacking, T-stacking, hydrophobic. Van der Waals interactions were excluded. The contacts are defined using the default geometrical criteria implemented in GetContacts [54]. In order to describe the mechanism of dissociation in the Fv and in the C_H_1–C_L_ region, we grouped together the residues belonging to the same loops or β-strands. The secondary structure has been assigned using STRIDE [55]. However, in the Fv region, the Kabat numbering scheme to identify the CDR loops has been respected (SI Fig. 4) [37]. The occurrence of the contacts during the US simulations has been reweighted using pyMBAR tool [53].

### Interdomain orientation

The mutual orientation between the V_H_–V_L_ domains is calculated using the ABangle tool [56]. It describes the orientation between the domains using five angles in degree, four bend angles (HC1, HC2, LC1, LC2) and one torsion angle (HL), plus a distance (dc) in Å. Following the same criteria, the OCD tool has been used to calculate the mutual orientations of the C_H_1–C_L_ domains, represented as well by four bend angles (cHC1, cHC2, cLC1, cLC2) and one torsion angle (cHL), and a distance (cdc) in Å [57].

## Results

The main aim of this work is to characterize one of the many determinants of protein thermostability, i.e. the domain–domain dissociation. Protein denaturation caused by high temperatures does not necessarily involve breaking of the covalent bonds in the polypeptide backbone, but rather the hydrogen bonds and non-covalent interactions tend to rearrange, leading to a less ordered state [58]. Also, the quaternary structure can be involved in this process, as dissociation or spatial rearrangement of the proteins’ subunits can occur. Here, we investigate the domain dissociation as one of the first steps of the melting process. To do this, we performed US simulations on 16 Fab structures to simulate their dissociation process. The systems in the dataset are the result of different LC–HC pairings, which are shown in Fig. 1a. Teplyakov et al. previously crystalized them and measured the melting temperature for each system [32]. The structures share the same constant region and the same CDR-H3 loop. The framework of the variable region and the other five CDR loops, instead, differ between the germlines.

The 16 Fabs break apart following two different pathways: the dissociation between the LC and HC can start in the Fv and continue in the C_H_1–C_L_ region or vice versa (SI Fig. 5). In both regions, the dissociation follows a two-steps mechanism: the bound structure evolves in an intermediate state that we name encounter complex and finally dissociates completely (SI Fig. 6). The identification of these three states is fundamental to characterize the dissociation process.

### Correlation between experimental data and computational results

The mechanistic understanding of the melting process in Fab structures is not clear yet. Differential scanning calorimetry (DSC) is one of the most common techniques to experimentally assess protein stability. In our simulation setup, instead of increasing the temperature, we applied a force that dissociates the two domains to provide a potential mechanism of the first steps of the melting process. Here, we make the assumptions that: (a) the domain dissociation simulated via US simulations can partially describe the process that the protein undergoes during melting; (b) the transition from the bound state to the encounter complex is a crucial contributor to thermal stability and thus can be used for its prediction. Therefore, for each system, we estimated the PMF curves resulting from the US simulations until this transition point, and we calculated the depth of the global minimum corresponding to the bound structure. The energy depth correlates with the melting temperatures, with a Pearson coefficient of 0.79 (Fig. 1b).

### Structural analysis of the dissociation

After proving the reliability of our computational results, we performed a structural analysis to highlight the residues that stabilize or destabilize the interface. Focusing on the paratope, the CDR-H3 sequence is conserved in all the structures, while the sequences of the other loops differ. Therefore, we compared the interactions of the LC CDR loops with the CDR-H3 loop. We found a high germline dependency when looking at the residues that stabilize the CDR-H3 loop.

#### Interactions of the CDR-H3 loop with the CDR-L3 loop

**Fig. 2:**
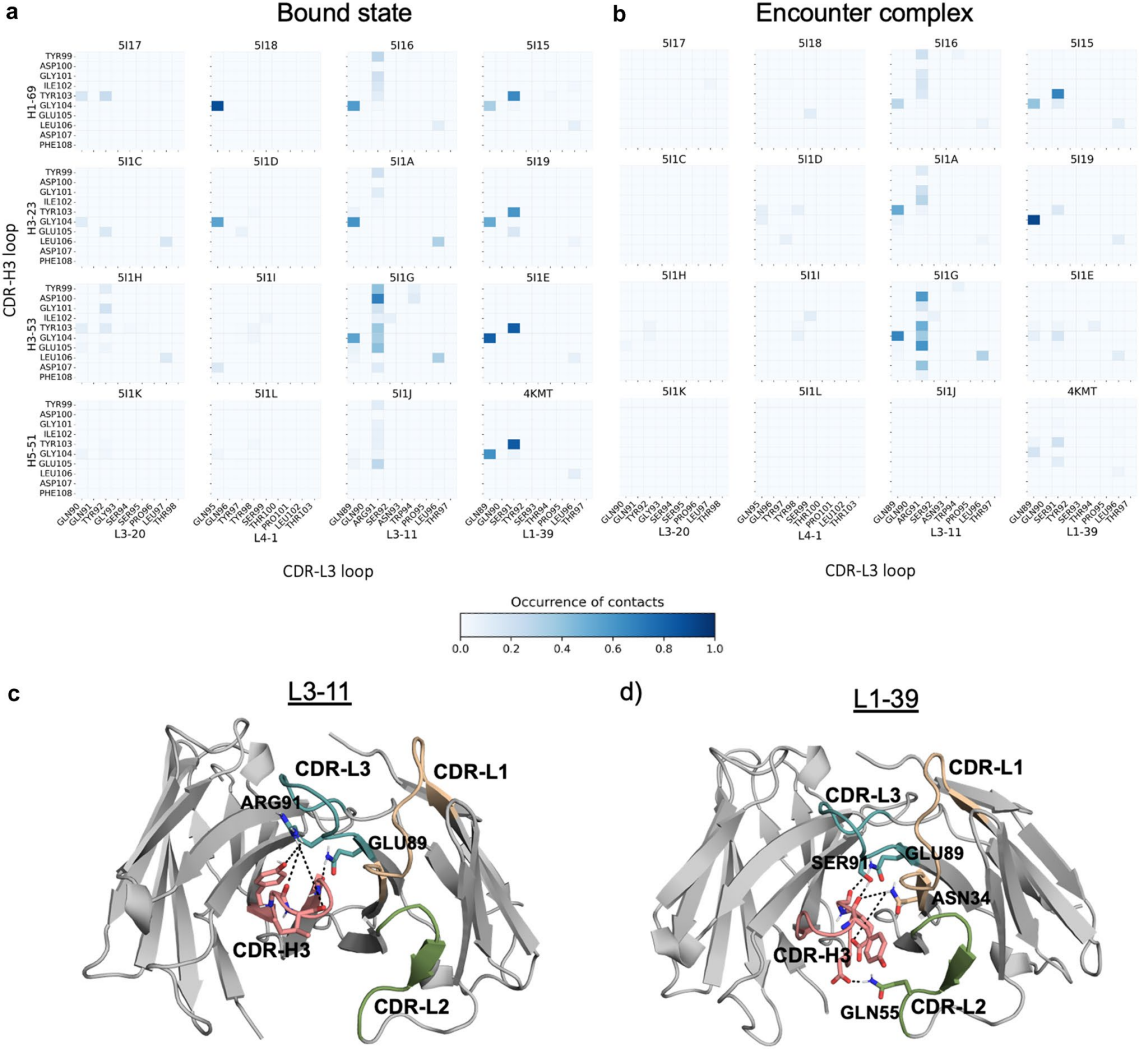
Interactions of the CDR-H3 loop with the CDR-L3 loop.** a** Occurrence of contacts between the CDR-L3 and the CDR-H3 loop in the bound state of each system. **b** Occurrence of contacts between the CDR-L3 and the CDR-H3 loop in the encounter complex of each system. **c** Top view of an exemplary Fv region comprising the L3-11 germline. The CDR-H3 loop is colored in light red, the CDR-L1 loop in light orange, the CDR-L2 in green and the CDR-L3 loop in light blue. The residues involved in loop interactions are illustrated as sticks and the contacts that they make are represented by black lines. Labels are shown for the residues in the CDR-L3 loop. **d** Top view of an exemplary Fv region comprising the L1-39 germline. The coloring follows the one in **d**. The residues involved in loop interactions are illustrated as sticks and the contacts that they make are represented by black lines. Labels are shown for the residues in the CDR-L3, CDR-L2 and CDR-L1 loops

Using the GetContacts tool [54], we calculated the occurrence of contacts that are present between the CDR-L3 and the CDR-H3 loop during the US simulations in the bound state and in the encounter complex for each system and reweighted it using pyMBAR [53, 54]. Figure 2a, and Figure 2b show the occurrence of contacts between the two loops respectively in the bound state and in the encounter complex. The residues in the CDR-L3 loop that interact with the CDR-H3 loop are mostly conserved between the four LC germlines, except the third residue of the CDR-L3 loop, which is the Tyr92 in the L3-20 and the Tyr97 in the L4-1 germlines, the Arg91 in the L3-11 germline and the Ser91 in the L1-39. The Arg91 in the L3-11 germline makes the highest number of contacts with several residues in the CDR-H3 loop (Fig. 2c). The CDR-L3/CDR-H3 contacts in the L1-39 germline occur more frequently than in the L3-20 and L4-1 germline. This is mainly because of the Gln89 and the Ser91 which make strong H-bonds with, respectively, the Gly104 and the Tyr103 in the CDR-H3 loop (Fig. 2d). Most of the CDR-L3/CDR-H3 contacts are H-bonds, apart from the Tyr92 (L3-20 germline)-Tyr103 which form a pi-stacking interaction. During dissociation, the occurrence of the contacts between the CDR-H3 and the CDR-L3 loop decreases (Fig. 2b). Some interactions are still present in the 5I16, 5I1A and 5I1G, thanks to the Gln89 and the Arg91 in the CDR-L3 loop of the L3-11 germline, and in the L1-39 germline system, between the Ser91 in the CDR-L3 loop and the Tyr103 in the CDR-H3 loop.

#### Interactions of the CDR-H3 loop with the CDR-L1 loop and the CDR-L2 loop

Also the CDR-L1 and CDR-L2 loops interact with the CDR-H3 loop, but in these cases the contacts are much less frequent compared to the CDR-L3/CDR-H3 loops interactions (up to 100% the occurrence of contacts in the bound state in the latter pair, compared to a maximum of 60% in the formers). The CDR-L1 loop shows low variability in sequence from one germline to the other, with the highest difference in the L4-1 germline, whose CDR-L1 loop is longer than in the other germlines. The same variability is also present in the CDR-L2 loops, however, in this case, the loop length stays constant in all germlines. The contacts between the CDR-H3 and the CDR-L1/CDR-L2 loops have been calculated and reweighted following the same protocol described above for the CDR-L3/CDR-H3 loops. SI Fig. 7a shows the CDR-L1/CDR-H3 contacts in the bound state of the structures. It is evident that the CDR-L1 loop in the germline L1-39 makes the highest number of contacts, in comparison to the other germlines, thanks to the contribution of the Asn34, which makes multiple H-bonds with the Tyr103, Gly104 and Glu105 in the CDR-H3 loop. Another relevant contact is the one between the Tyr32 in the CDR-L1 loop and the Tyr103 in the CDR-H3 loop. This contact is also present in the other LC germlines, but with a much lower occurrence. In the dissociation process, the CDR-L1/CDR-H3 contacts are only present in the system derived from the L1-39 germline, in which the Asn34 still strongly interacts with the CDR-H3 loop (SI Fig. 7b). The CDR-L2 loop, instead, makes less interactions with the CDR-H3 loop (SI Fig. 7c). Quite occurrent is the contact between the Gln55 in the CDR-L2 loop of L1-39 germline and the Asp107 in the CDR-H3 loop. However, this interaction is almost not visible anymore during dissociation (SI Fig. 7d).

#### C_H_1–C_L_ interdomain contacts

**Fig. 3:**
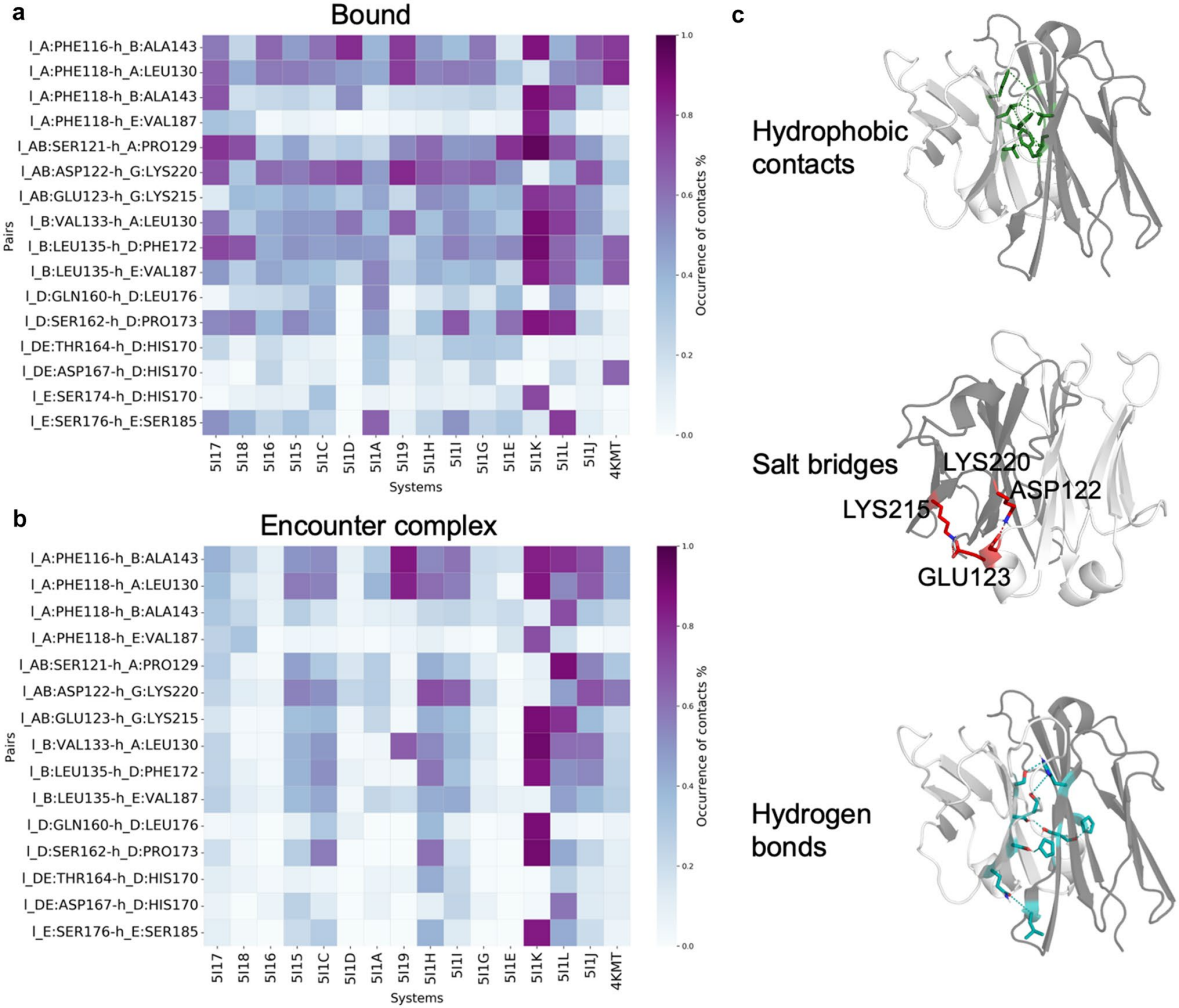
C_H_1–C_L_ interdomain contacts.** a** Representation of the occurrence of the contacts in the bound state of the simulations. The residues that make contacts are shown in the y axis: the first residue belongs to the C_L_ and the second one to C_H_1. The capital letters represent the strands or loops where these residues are located. **b** Representation of the occurrence of the contacts in the encounter complexes. **c** Stick representation of the paired residues in the C_H_1–C_L_ interface, with specification of the type of contacts that they make. The light grey domain is the C_L_, the dark grey is the C_H_1

All the germlines share the same constant region. Figure 3a shows the occurrence of the contacts in the bound state of the systems. Only the contacts with an occurrence greater than 0.3 are shown. The numeration of the constant region is described in SI Fig. 8. Most of the contacts are H-bonds or hydrophobic ones. Only the Asp122 and the Glu123 in the C_L_ make a salt bridge respectively with the Lys220 and the Lys215 in the G strand of the C_H_1 (Fig. 3c). A lysine is also present in the G strand of the LC, in position 207. However, it does not have any negatively charged partner in the opposite chain to establish a salt bridge. Seldomly, the Lys207 in the LC makes H-bonds with several residues in the AB strand of the HCs. These contacts are not shown in Fig. 3, because their occurrence is low. Leu135 in the LC has also an important role: thanks to its central position, it makes several hydrophobic contacts with strand B (Ala143), D (Phe172) and E (Val187) in the HC. Generally, the C_H_1–C_L_ interdomain contacts still take place in the encounter complex, even if with a lower occurrence compared to the bound state (Fig. 3b).

### Mechanism of dissociation in the Fv

**Fig. 4:**
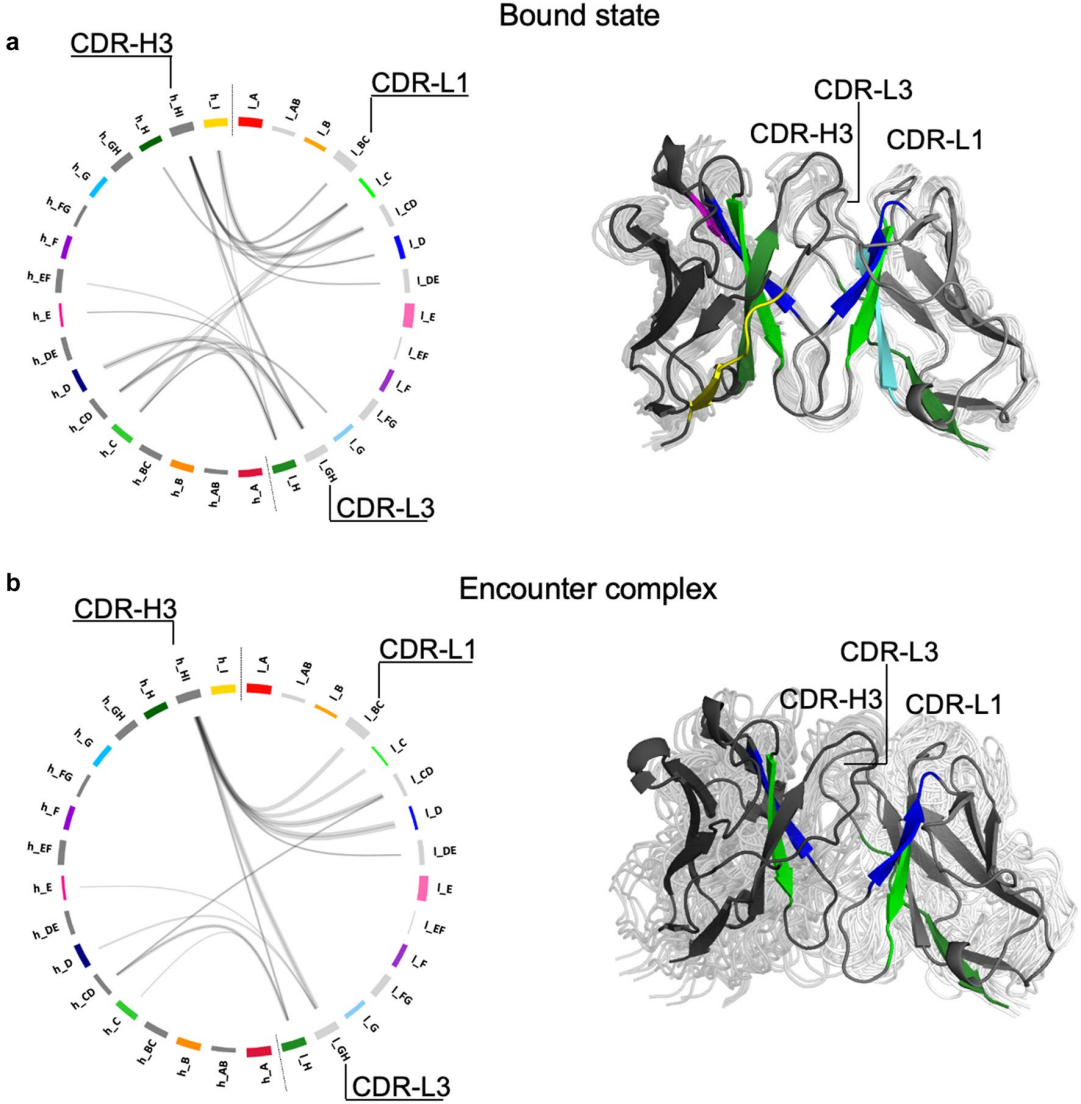
Mechanism of dissociation in the Fv region. Exemplary representation of the contacts in the Fv region in the bound state (**a**), at the bound–encounter transition point (**b**). On the left side, the contacts are represented by flareplots. On the right side, a structural representation of the state: in the background, the ensemble of conformations that an exemplary structure adopts in the selected timeframes; in front, a cartoon representation of the structure at the first frame of each timespan. The β-strands involved in interdomain contacts are colored according to the flareplots. The light chain (in light grey) is on the right side, the heavy chain (in dark grey) is on the left

Generally, the US simulations lead to dissociation in both regions (Fv and C_H_1–C_L_) at different points in time. Only for two systems (5I1C and 5I1J) the dissociation is only observed in the Fv region (SI Fig. 5). A partial dissociation and a rearrangement of the C_H_1–C_L_ domains of these two systems is observable in later stages of the pulling simulations, but this event is not visible in the selected windows (maximum distance between the COMs of the LC and HC equal to 4 nm). We represent the interdomain contacts with the so-called flareplots, grouping the residues belonging to the same strand or loop (SI Fig. 4). To describe how the interdomain contacts develop during the dissociation process, we consider snapshots of 2 ns starting from the beginning of the US simulations, and from the bound–encounter transition points. This timeframe can give a good statistic of the contacts that take place at the beginning of the simulation and in the transition state. We neglect the encounter–unbound transition because the unbound state is not relevant for our study. Figure 4 shows an exemplary representation of the interdomain contacts that are present in one system in these timeframes. The result stays generally consistent for all the systems, with few differences observed. Focusing on the Fv region, the bound structure shows multiple contacts between the β-strands h_C, h_D, h_E, h_H, h_I and the neighboring loops in the HC and the β-strands l_C, l_D, l_G and l_H and the neighboring loops in the LC (Fig. 4a). At the transition point from bound to encounter complex, some contacts are already missing. The CDR-H3 loop (h_HI) still makes contacts with the CDR-L1 (l_BC) and the CDR-L3 (l_GH) loops (Fig. 4b). Some β-strands are still involved in interdomain contacts at this point of dissociation, but these contacts can evolve quite fast and can differ from one system to the other, depending on the domain orientation.

### Mechanism of dissociation in the C_H_1–C_L_ region

**Fig. 5:**
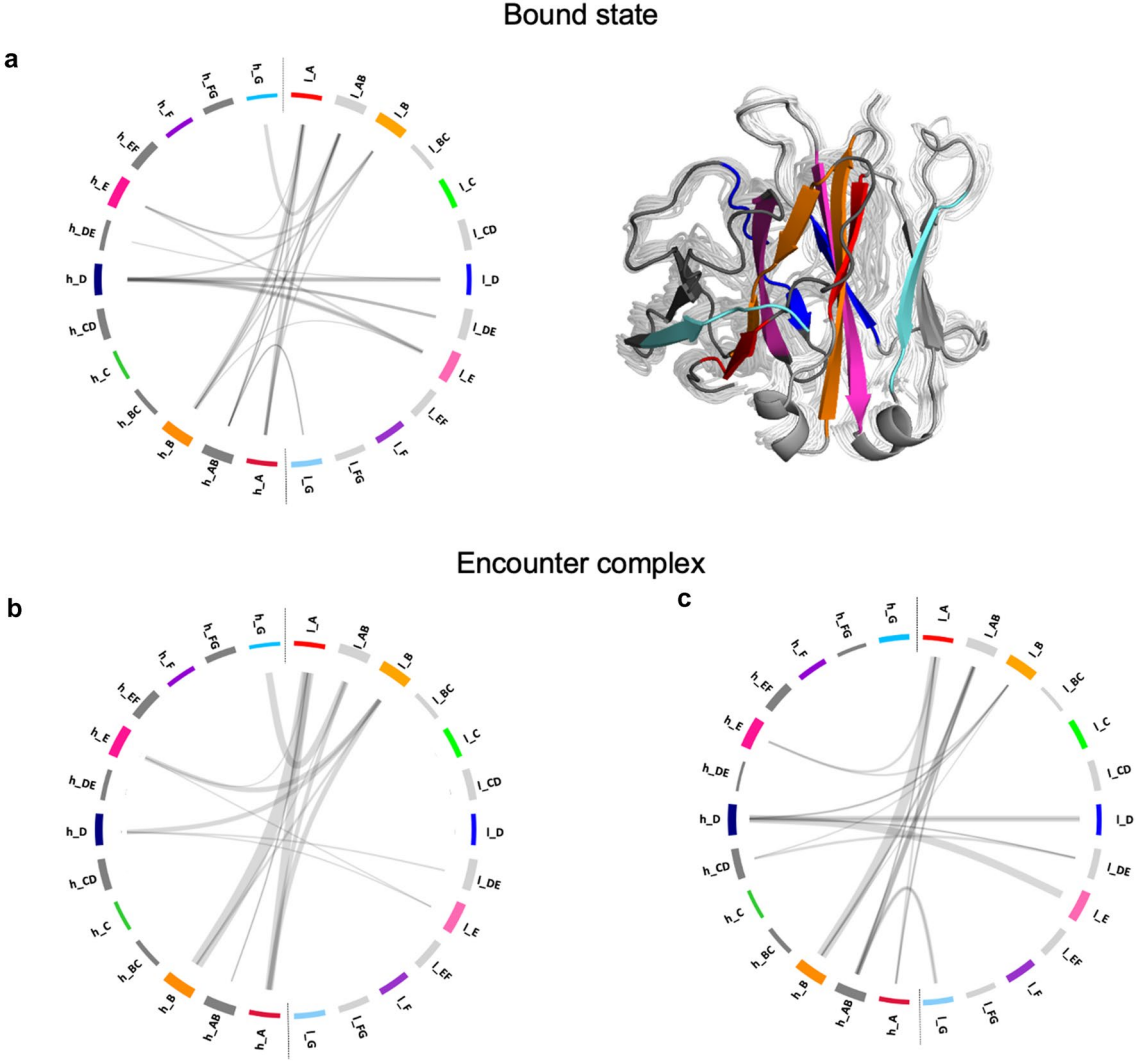
Mechanism of dissociation in the C_H_1–C_L_ region. Representation of the contacts in the C_H_1–C_L_ region in the bound state (**a**) and at the transition point from the bound state to the encounter complex (**b**,** c**). A structural depiction of the bound state is also provided: in the background, the representation of the ensemble of structures; in front, a cartoon representation. The β-strands involved in interdomain contacts are colored according to the flareplots. The light chain (C_L_, in light grey) is on the right side, the heavy chain (C_H_1, in dark grey) is on the left. The bound–encounter transition can happen following two mechanisms depicted in **b** and **c**

In order to compare different interfaces, we repeated the same procedure as for the Fv region also for the C_H_1–C_L_. We plotted the contacts that exist in the C_H_1–C_L_ interface, showing flareplots in which the residues that belong to the same loops or β-sheets are grouped together (SI Fig. 4). Also for this region, we looked at the contacts that are present in the C_H_1–C_L_ interface considering snapshots of 2 ns at the beginning of the US simulations and at the bound–encounter transition point. Figure 5 shows the interdomain contacts in a representative system during dissociation. The findings are transferable also to the other systems with minor differences. The bound state is characterized by contacts that involve the β-strands A, B, D, E and G and the loops AB and DE in both chains (Fig. 5a). When the structure transits from a bound state to the encounter complex state, it can adopt two different conformations: either the h_G strand interacts with the l_AB loop (Fig. 5b), or l_G interacts with h_AB (Fig. 5c). In any case, the G strand of one of the two chains is the first element to lose contacts with the opposite domain. The only two salt bridges that are present in the C_H_1–C_L_ structure are formed between h_G and l_AB (Fig. 3). In the dissociation pathway shown in Fig. 5c, these salt bridges are missing and the whole structure is kept together only by hydrophobic interactions and H-bonds.

### Shifts in the interdomain orientation during dissociation

We analyzed how the angles that describe the movement in the interface change during the dissociation process. SI Fig. 9a shows the shifts in the V_H_–V_L_ interface angles for each system until the end point of dissociation, calculated with the ABangle tool [56]. The standard deviation of each angle during this time has also been calculated and it is shown in each plot. In four systems (5I17, 5I1A, 5I1H and 5I1K) the biggest shift is present in the HL angle. For all the other systems, instead, the bend angles are the ones that change the most during dissociation. The same analysis has been done also for the C_H_1–C_L_ region, using the OCD tool [57] (SI Fig. 9b). Excluding the 5I1C and the 5I1J that don’t dissociate in the C_H_1–C_L_ region during our simulations, only three systems show a bigger shift in the torsion angle (5I15, 5I19, 5I1K). In most of the cases, instead, the dissociation causes a change of the bend angles.

## Discussion

In this work we structurally characterize the mechanism of dissociation of 16 Fab fragments [32]. This dataset has been chosen because of the availability of experimental data, such as crystal structures and melting temperatures. The melting temperatures have been measured using DSC, one of the most common and efficient techniques to assess protein melting and unfolding, which provides the difference in temperature between a reference and a sample cell [59]. DSC remains unparalleled to assess the thermodynamic stability of proteins [60] and this type of measurement is particularly relevant for antibodies because stability directly impacts their structure and ultimately their function [61, 62]. Thermal unfolding involves loss of tertiary structure, followed by loss of some elements of secondary structure, and general unfolding of the protein [63]. The profile of the temperature-induced unfolding for a whole IgG1 mAb consists of two peaks: the high-temperature peak comprises an overlay of two transitions—reversible unfolding of C_H_2–C_H_2 domains followed by irreversible unfolding of the Fab-, instead the low-temperature one displays the melting of the C_H_3–C_H_3 domains [64–67]. Even if the melting process of the whole antibody molecule has been thoroughly studied, the mechanism of unfolding of the domain complexes at the interface level is still unknown. The interface between the domains is stabilized by non-covalent interactions, that are highly susceptible to stress at high temperatures. Therefore, the quaternary structure of the protein will potentially be the first one to be affected in the melting process, resulting in domain dissociation or spatial rearrangement. In antibody design, this is particularly relevant because chain mispairings or non-favorable orientations of the domains can strongly modify the antigen binding site, resulting in a non-functional antibody [68]. For this reason, several studies have been carried out to mutate the interface residues and, on one side, ensure the correct chain pairing, but also increase the overall protein stability [4, 19]. It is therefore important to characterize the interactions that take place in the antibodies’ interface, to avoid functionality or developability issues [69].

In order to address this open question and simulate the domain dissociation, we ran US simulations, which overtake the sampling limits of MD simulations. This enhanced sampling technique is established to study protein–protein binding processes or to assess protein stability [52, 70]. Another advantage of US is the possibility to obtain a reweighted free energy curve that describes the process of interest.

In our simulation, we observe that the bound structure evolves to an encounter complex state, before completely dissociating (SI Figs. 5, 6). However, while the bound state and the encounter complex are well defined, the unbound state shows the highest diversity and thus uncertainty. Therefore, we assume that the melting process represents the transition between the bound state and the encounter complex. The depths of the minima of the free energy curves, that correspond to the energy of the bound state, are compared to the experimentally measured melting temperatures, to assess the reliability of our computational results. The correlation shows that, even if we are neglecting other important aspects connected to thermal stability, the domain dissociation process captured by our simulations represents a critical step in the melting process (Fig. 1b). The analysis of the free energy of the bound states can properly separate the stable from the unstable Fabs. However, systems with the same melting temperature can result in different values of energy associated to the bound state and some outliers can be identified. This is mainly due to the simplification of the highly complex melting process to the domains’ dissociation. Moreover, the dissociation happens in both Fv and C_H_1–C_L_ regions for 14 systems out of 16. In the Fabs with PDB codes 5I1C and 5I1J, instead, only a full dissociation in the Fv region is visible. This is because of the cutoff that we chose before starting US simulations (4 nm COM distance between LC and HCs, see Methods). For these two systems, in fact, the C_H_1–C_L_ dissociation starts only at a later stage in the pulling simulations.

Antibodies’ biophysical properties are highly influenced by the germlines and their respective HC and LC pairings [71]. The overall stability depends on the intrinsic structural stability of each domain, as well as on the extrinsic stabilization provided by their interaction [72]. Our 16 Fab structures are the result of different pairings of four HC germlines with four LC germlines (all κ) and, therefore, we were able to derive how different germline pairings influence the Fab stability.

The Fv region, and especially the paratope, often causes instability because of the high variability in sequences and conformations. On the other hand, the loop region couples with the protein core and substantially contributes to the stability of the fold [21]. The germlines in our dataset differ in CDR loops length and sequence, except for the CDR-H3 loop which is conserved. Even if all the loops contribute to shape the paratope, the CDR-H3 loop raises the main interest because it makes on average the highest number of contacts with the antigen [73–75]. The contacts between the V_L_-CDR loops and the CDR-H3 loop provide a meaningful stabilization of the variable region. In particular, the CDR-L3 and CDR-H3 loops lie together in the center of the antigen binding site and are relevant to shape the paratope [16]. Besides, they have comparable diversity in sequence, length and structure [76–78]. We compared the effect of CDR loops derived from the different germlines on the same CDR-H3 loop. Our findings suggest that the distinct LC–HC pairings highly influence the stability of the Fab structures. Comparing the CDR loops interactions in the different systems, it is evident that the LC germlines present in the most stable Fabs (L3-11 and L1-39) are also the ones in which the paratope is highly stabilized. Focusing on the CDR-H3/CDR-L3 interactions, when an Arg is present as third residue of the CDR-L3 loop, as in the germline L3-11, it forms more interactions with the CDR-H3 loop rather than when a Tyr or a Ser is in the same position (Fig. 2a). As previously discussed in literature, Arg located in the CDR loops, is a critical determinant for affinity [79–81]. In this case, it seems that this residue may play an important role in the stabilization of the CDR-H3 loop, too.

The CDR-L1 and the CDR-L2 loops also interact with the CDR-H3 loop, even if their contacts are less occurrent than the ones between CDR-L3 and CDR-H3 loop (SI Fig. 7). In our dataset, CDR-L1 loops show quite some differences in sequence and length, whereas CDR-L2 loops share the same length but differ in sequence. Loop length variation is proven to be a valid strategy for affinity maturation of antibodies, since it influences tertiary structure and activity simultaneously [82, 83]. However, an enhancement in affinity does not always go hand in hand with a higher stability. In our dataset, for example, the two germlines with longer CDR-L1 loops, L3-20 and L4-1 (respectively 12 and 17 AA) show only few stabilizing contacts with the CDR-H3 loop. Instead, the L1-39 germline, which is also the most stable one, makes the highest number of interactions (SI Fig. 7a). The interactions between CDR-L1 and CDR-H3 loops can mainly be attributed to the Asn34, i.e., the last residue of the CDR-L1 loop in the L1-39 germline, whose contacts with the CDR-H3 loop are still present in the encounter complex (SI Fig. 7b). The presence of the Asn in the variable region can lead to a degradation mechanism, i.e., deamidation, especially when it adopts particular geometries on the tip of a loop [84]. The Asn34 in this case, though, can be highly stabilized by the neighboring Trp35 present in the framework [85].

The CDR loops can adopt several conformations in solution that result in different interactions [75, 86] and this is also confirmed in this work. In fact, the contacts that the CDR loops make in the encounter complexes are not always the same as in the bound state, suggesting a conformational rearrangement of the loops. This is evident, for example, in the CDR-L3/CDR-H3 interactions in the 5I1I, 5I1A and 5I1E (Fig. 2b), where different loops interactions are present in the encounter complex compared to the bound state. Even if a rigid interface is usually associated to a specificity in the binding [10, 87, 88], backbone flexibility allows conformational adjustments that can lead to stabilizing, low-energy interactions [89].

Interdomain contacts are key determinants for stability. Therefore, we studied the constant domain interface, showing that it is mainly characterized by hydrophobic interactions and H-bonds [69]. The β-strands A, B, D, E and the loops AB and DE in both chains are mainly responsible for the interdomain interactions, since they are in the center of the interface (Fig. 3). The only two salt bridges in the C_H_1–C_L_ interface are formed between two lysines (Lys215, Lys220) in the β-strand G in the HC and the AB loop in the LC. Interestingly, the β-strand G is the first strand to lose its contacts with the partner domain during dissociation (Fig. 5). The encounter complex state, in which the protein–protein interface is partially solvated and contains non-optimal sidechain orientations, is characterized mainly by hydrophobic interactions [90]. Not only the C_H_1–C_L_ region provides a further stabilization of the Fab fragment [24], but also the latest development in antibody engineering introduced modifications in the C_H_1–C_L_ interface, obtaining stabilized heterodimeric interfaces, to facilitate the production of bispecific antibodies [31, 68, 91, 92]. Therefore, the study of the interactions in the first constant domain interface is highly relevant for the design of novel therapeutics.

Furthermore, we found exemplary mechanisms of dissociation in the Fv and in the C_H_1–C_L_ region. In our simulations, we do not sample the unfolding of the single domains, which happens only after the loss of contacts at the interface between the V_H_–V_L_ and C_H_1–C_L_ domains [93]. The Fab structures that compose our dataset dissociate in the Fv region and in the C_H_1–C_L_ at different times (SI Fig. 5), showing high cooperation between the two interfaces [94]. The analysis of the dissociation process in the Fv region shows that several contacts are present between the two domains in the bound state, but especially the interactions between the CDR-H3 and the CDR-L3 loop keep the structures together until complete dissociation (Fig. 4). The dissociation in the C_H_1–C_L_ region, instead, is characterized by a reorientation of the two domains that leads to a loss of the contacts of the G strand (Fig. 5b, c).

A key aspect in antibody modelling and engineering is the domain orientation, since it highly influences the binding mechanism and the overall affinity [56]. The dissociation affects, of course, the orientation between the LC and the HC. The separation of the two domains can be mainly represented by big shifts in the bend angles, in both the Fv and the C_H_1–C_L_ region, whereas only few systems show major changes in the HL angle (SI Fig. 9).

## Conclusion

Low thermal stability is one of the main issues in rational antibody design. We studied the stability of 16 Fab structures, characterizing their mechanism of dissociation. The paratope stabilization and the interdomain contacts are key determinants for the Fab stability. Our results show that the contacts between the CDR loops are highly germline dependent and especially the ones between the CDR-H3 and the CDR-L3 loop occur during the whole dissociation process, stabilizing the paratope. On the other hand, the Fv and the C_H_1–C_L_ interface show co-operativity in the stabilization of the Fab. The hydrophobic interdomain contacts and the H-bonds located in the core of the constant domain interface occur during the whole dissociation process, additionally stabilizing the Fab.

## Supplementary Information

Below is the link to the electronic supplementary material.

Supplementary file1 (DOCX 21638 KB)

## Data Availability

The authors confirm that the data supporting the findings of this study are available within the article and its supplementary materials.
