# Supplementary material for: Structural mechanism of Fab domain dissociation as a measure of interface stability
Source: J Comput Aided Mol Des. 2023 Mar 15;37(4):201–15. doi: 10.1007/s10822-023-00501-9 (PMC10049950; doi:10.1007/s10822-023-00501-9)
Supplement: Supplementary file 1 — Supplementary file1 (DOCX 21638 KB) [file 10822_2023_501_MOESM1_ESM.docx]

# **Structural Mechanism of Fab Domain Dissociation as a Measure of Interface Stability**

Nancy D. Pomarici^1^, Franz Waibl^1^, Patrick K. Quoika^1,2^, Alexander Bujotzek^3^, Guy Georges^3^, Monica L. Fernández-Quintero^1^*, Klaus R. Liedl^1^*

*^1^Institute of General, Inorganic and Theoretical Chemistry, and Center for Molecular Biosciences Innsbruck (CMBI), University of Innsbruck, Innrain 80-82, 6020 Innsbruck, Austria.*

*^2^Center for Protein Assemblies (CPA), Physics Department, Chair of Theoretical Biophysics, Technical University of Munich, Ernst-Otto-Fischer-Str. 8, 85748, Garching, Germany*

*^3^Roche Pharma Research and Early Development, Large Molecule Research, Roche Innovation Center Munich, Nonnenwald 2, 82377, Penzberg, Germany.*

*✉ email:* [*monica.fernandez-quintero@uibk.ac.atmonic*](mailto:monica.fernandez-quintero@uibk.ac.at)*, Klaus.Liedl@uibk.ac.at*

**
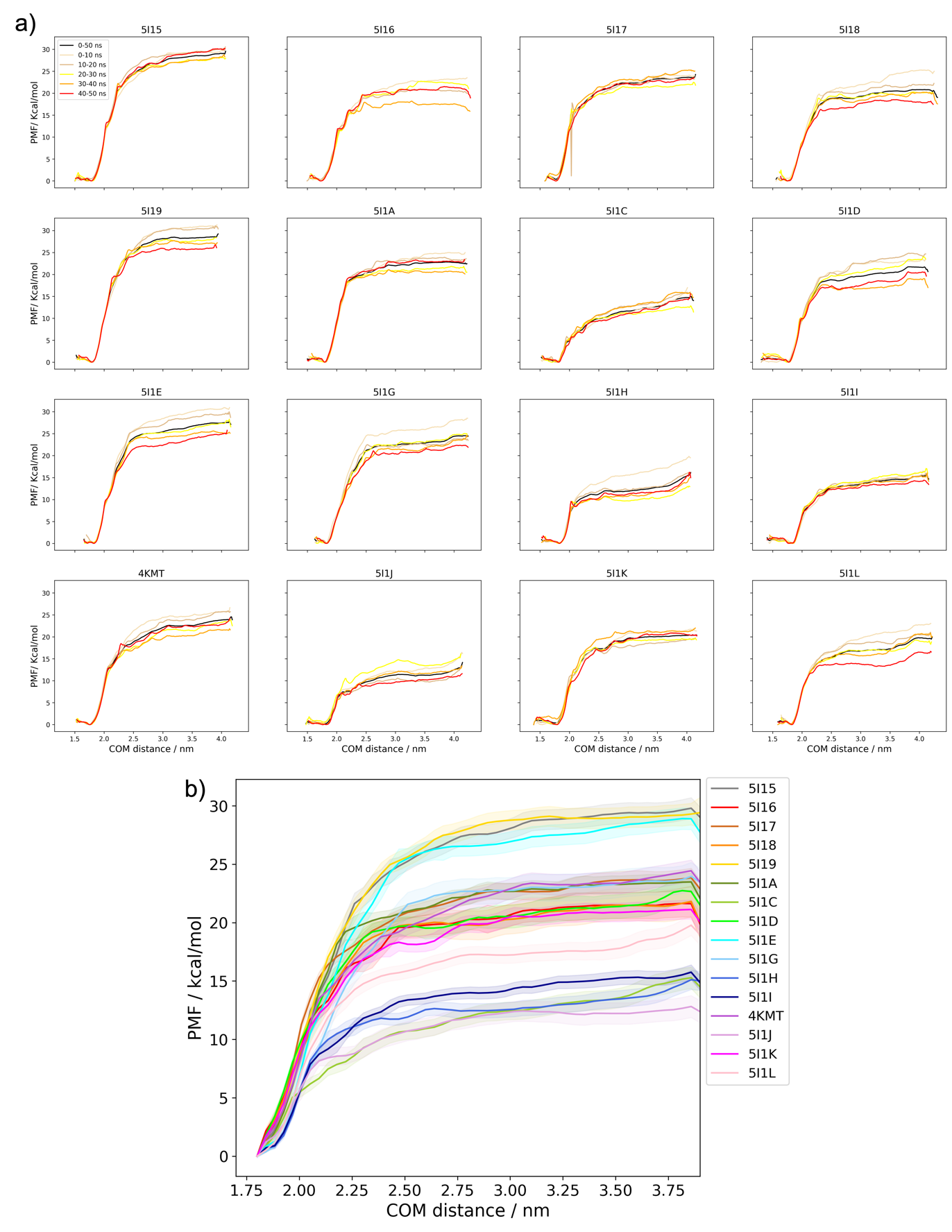
SI Fig.1. PMF curves of the US runs of each system. a)** To assess the convergence of the US runs, the US windows are splitted into 5 segments of 10 ns each and the PMFs are calculated for each fragment. The results for each system are shown. In wheat the 0-10 ns fragment PMF curve is shown, in light brown the 10-20 ns, in yellow the 20-30 ns, in orange the 30-40 ns, in red the 40-50 ns and in black the overall 50 ns PMF curve. **b)** The reweighted PMF curves of each system are colored according to the legend. The error associated to each curve is shown in transparent.

**
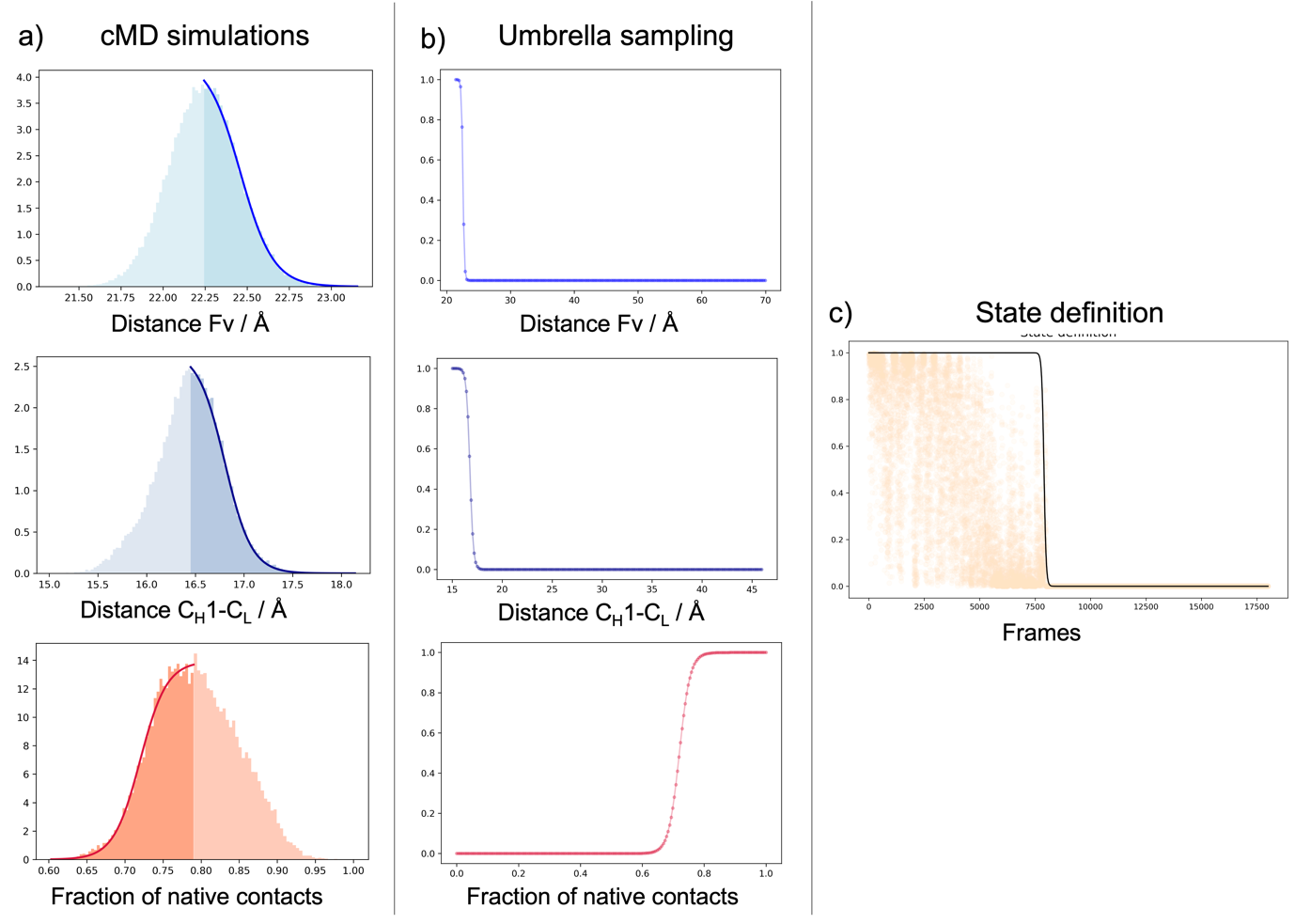
**

**SI Fig.2. Transition point from the bound state to the encounter complex. a)** Representation of the bell-shaped distribution of the distances and fraction of native contacts during the cMD simulations, used as descriptors to define the dissociation points in the Umbrella Sampling (US) simulations. A decreasing sigmoidal function is fitted on the right side of the distance distributions and an increasing one is fitted on left side of the fraction of native contacts distribution. **b)** The parameters of the previous functions are used to plot new sigmoids on the values of distances and fraction of native contacts that are sampled during the US simulations. These sigmoids approach 1 when the structure is still bound, and they approach 0 when the dissociation is starting. **c)** State definition obtained by multiplication of the previous three sigmoids. It shows the state of the system over time, starting from a bound structure (corresponding to 1), towards a structure that starts dissociating, namely the encounter complex (0). A new sigmoidal function can be fitted to reduce the noise. The point at which the sigmoid approaches 0 can be estimated and this corresponds to the transition from the bound state to the encounter complex.

**
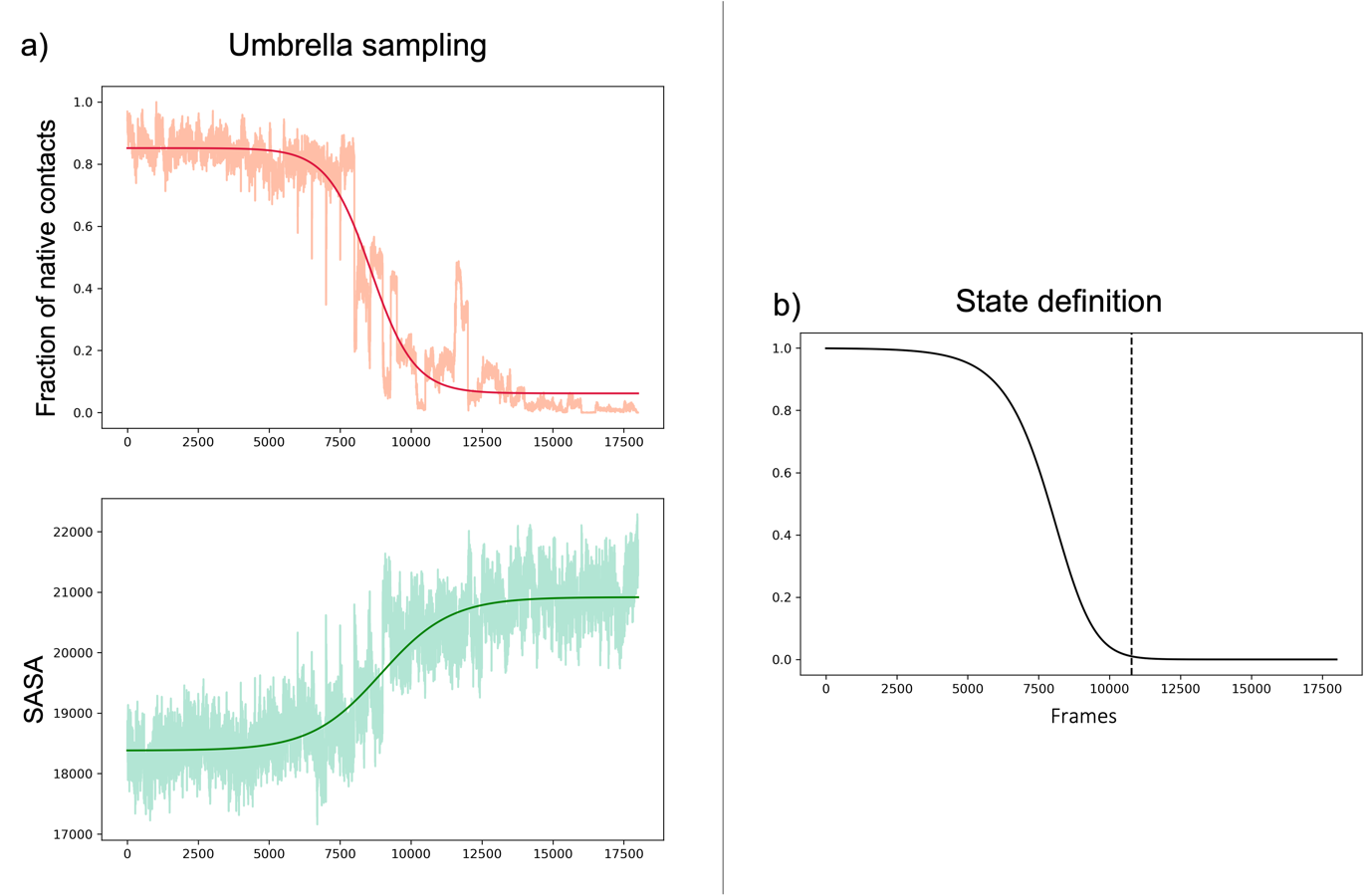
**

**SI Fig.3. Transition point from the encounter complex to the unbound state. a)** Timeseries of the fraction of native contacts and SASA during the US simulation. The timeseries can be approximated by two sigmoids. **b)** Sigmoidal function representing the state definition of the system. It is obtained by normalization and multiplication of the two sigmoids shown in **SI Fig.3a**. The state 1 represents the bound state and 0 represent the unbound one. The frame at which the sigmoid approaches 0 is considered the end point of dissociation.

**
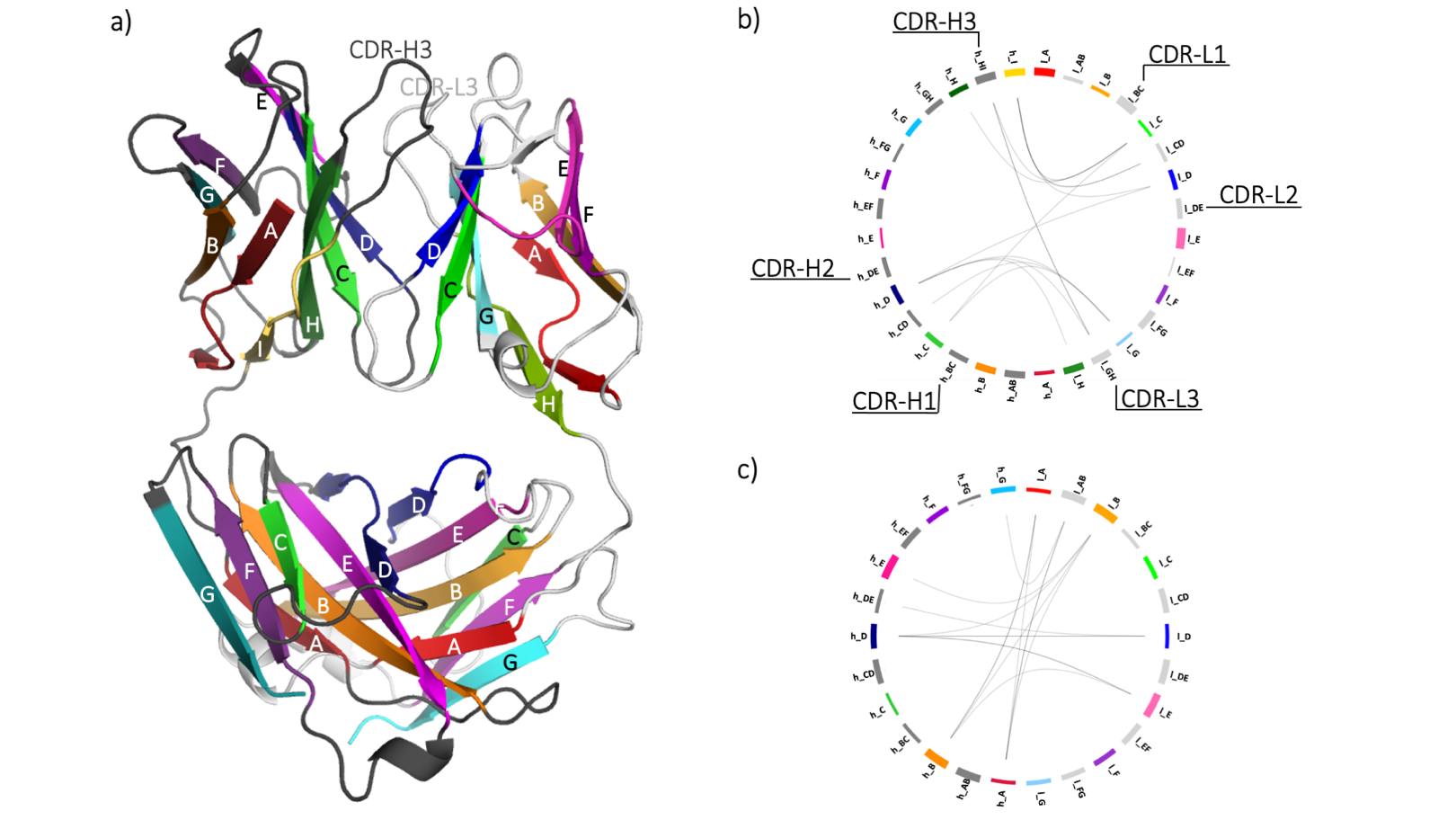
**

**SI Fig.4. Grouping of the secondary structure elements. a)** Schematic representation of a Fab structure, grouping the residues that belong to the same loop or β-strand. The light chain is on the right side and the heavy chain on the left. The loops in the light chain are colored in light grey, whereas the ones in the heavy chain are in dark grey. **b)** Example of flareplot showing the contacts at the interface between the V_H_ (on the left side) and V_L_ (on the right side) domains. The residues belonging to the same secondary structure element are grouped together and color coded according to the structure in **SI Fig.4a**. The letters representing each loop or β-strand are preceded by the letter “h_” if they belong to the heavy chain or “l_” if they are in the light chain. **c)** Example of flareplot showing the contacts at the interface between the C_H_1 (on the left side) and C_L_ (on the right side) domains. The residues belonging to the same secondary structure element are grouped together and color coded according to the structure in **SI Fig4a**. The letters representing each loop or β-strand are preceded by the letter “h_” if they belong to the heavy chain or “l_” if they are in the light chain.


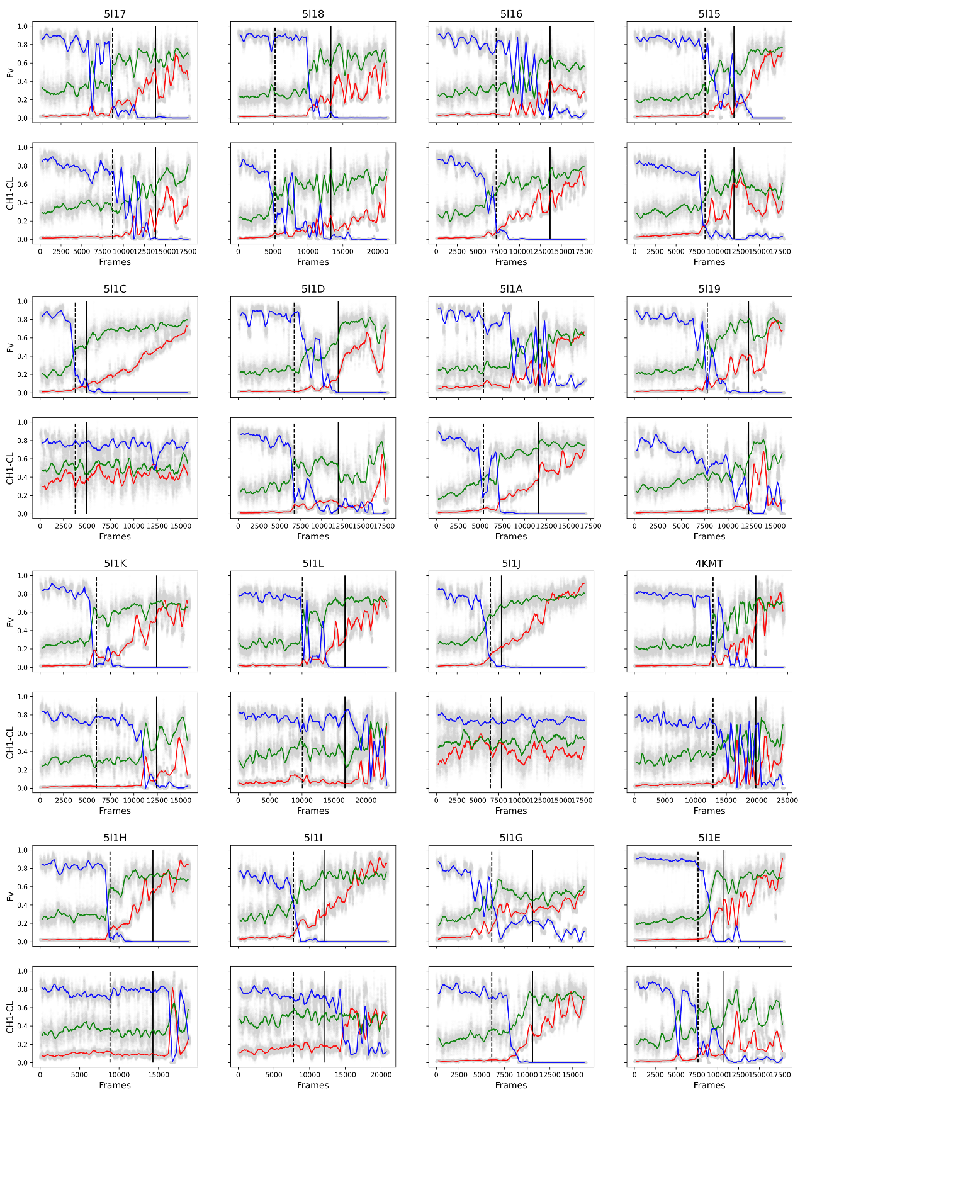


**SI Fig.5. Dissociation in the Fv and in the C_H_1-C_L_ region.** Timeseries of the distance between the COM of the two domains (red line), fraction of native contacts (blue) and SASA (green) in the Fv region (upper plots) and in the C_H_1-C_L_ region (lower plots). The dashed and the continuous vertical lines indicate respectively the transition point from the bound state to the encounter complex and from the encounter complex to the unbound state.


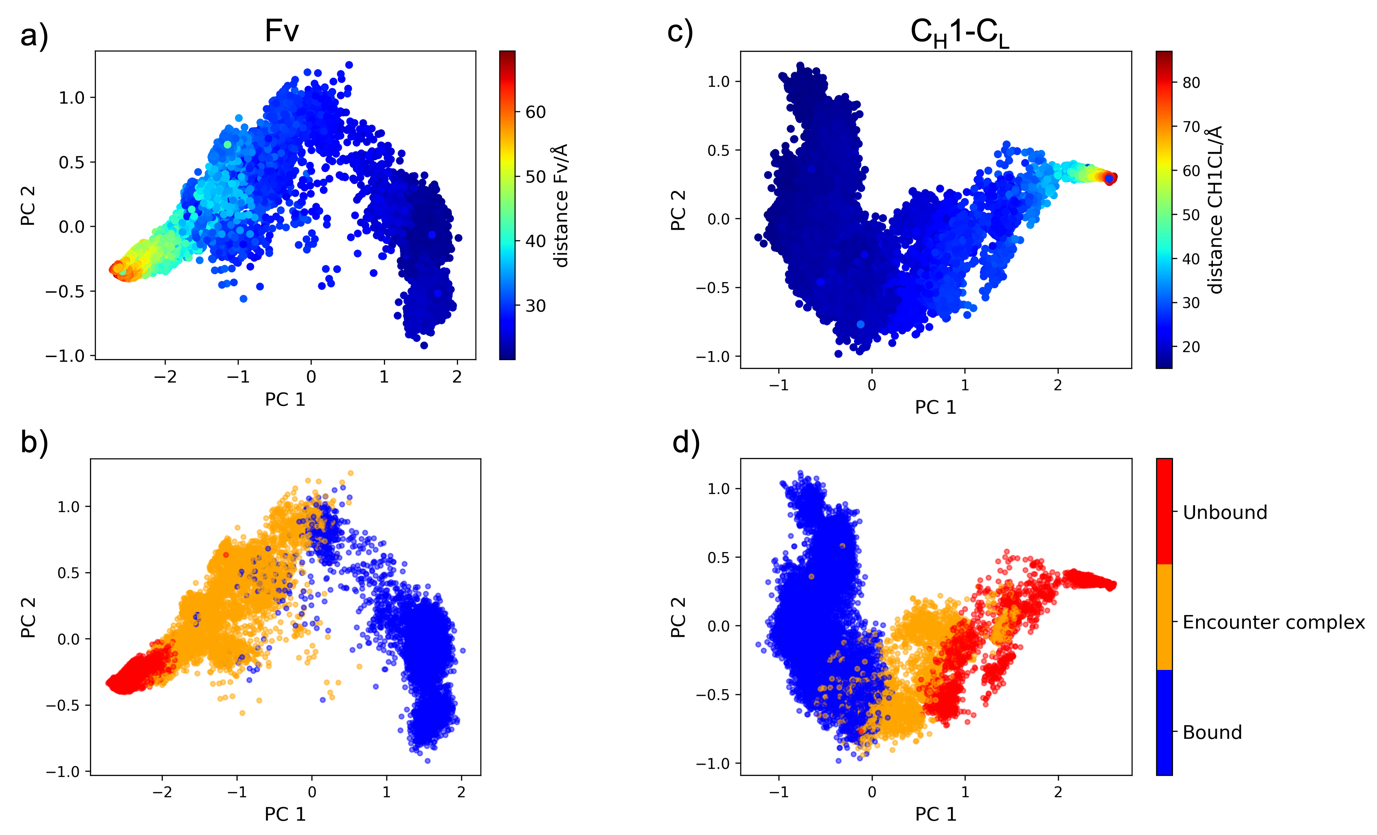
**SI Fig.6. Two-steps dissociation mechanism. a)** Principal components analysis (PCA) describing the dissociation process in the Fv region for of one exemplary system (PDB code: 5I19). The PCA uses as features the inverse distances between the residues that make contacts in the starting structure in the Fv region. The values of distance between the COM of V_H_ and V_L_ is plotted in the PCA space. **b)** States separation during the dissociation process in the Fv region. **c)** Principal components analysis (PCA) describing the dissociation process in the C_H_1-C_L_ region for one exemplary system (PDB code: 5I19). The PCA uses as features the inverse distances between the residues that make contacts in the starting structure in the C_H_1-C_L_ region. The values of distance between the COM of C_H_1 and C_L_ is plotted in the PCA space. **d)** States separation during the dissociation process in the C_H_1-C_L_ region.

**
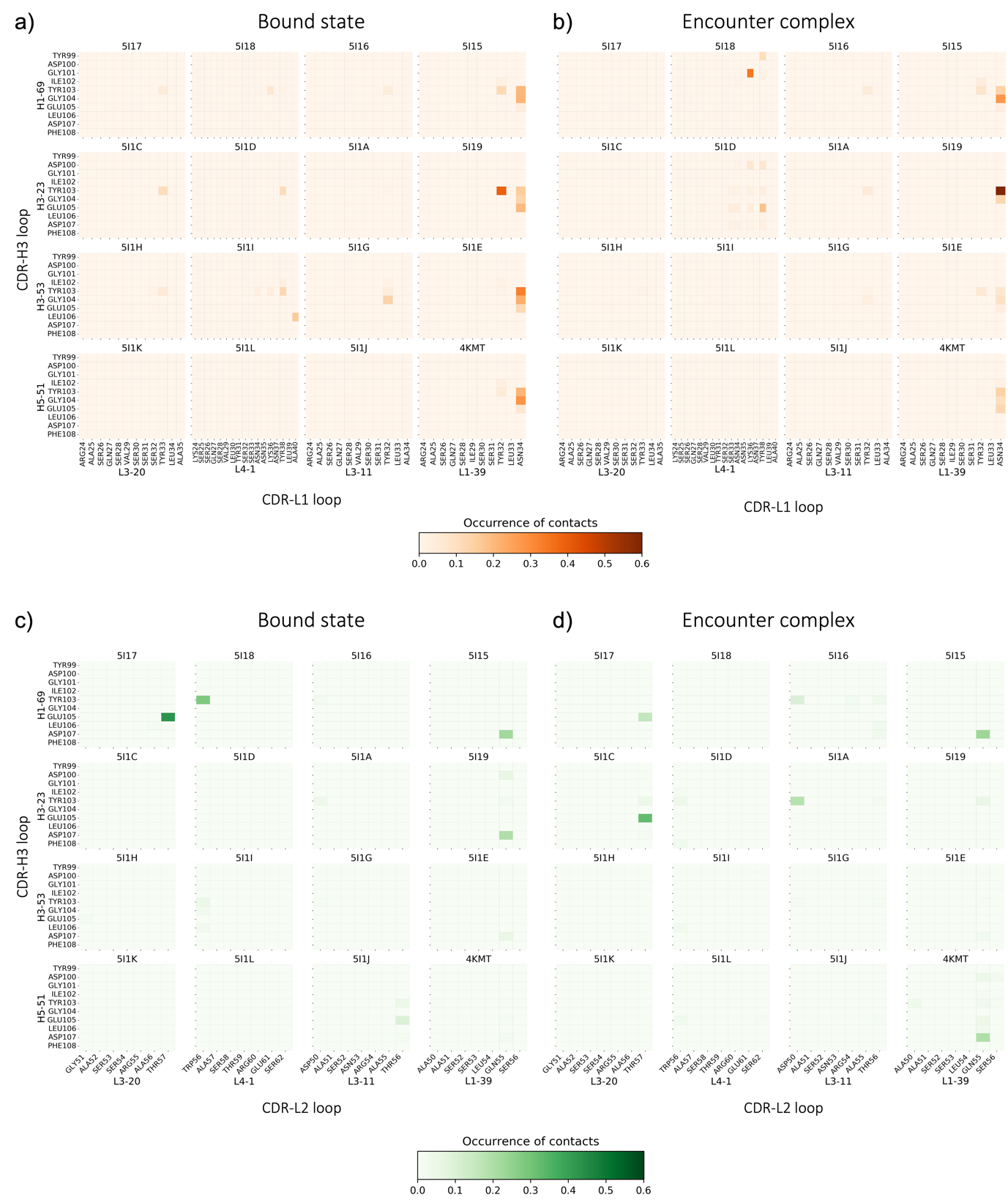
SI Fig.7. Interactions of the CDR-H3 loop with the CDR-L1 and CDR-L2 loops.** **a)** Occurrence of contacts between the CDR-L1 and the CDR-H3 loop in the bound state of each system. **b)** Occurrence of contacts between the CDR-L1 and the CDR-H3 loop in the encounter complex of each system. **c)** Occurrence of contacts between the CDR-L2 and the CDR-H3 loop in the bound state of each system. **d)** Occurrence of contacts between the CDR-L2 and the CDR-H3 loop in the encounter complex of each system.


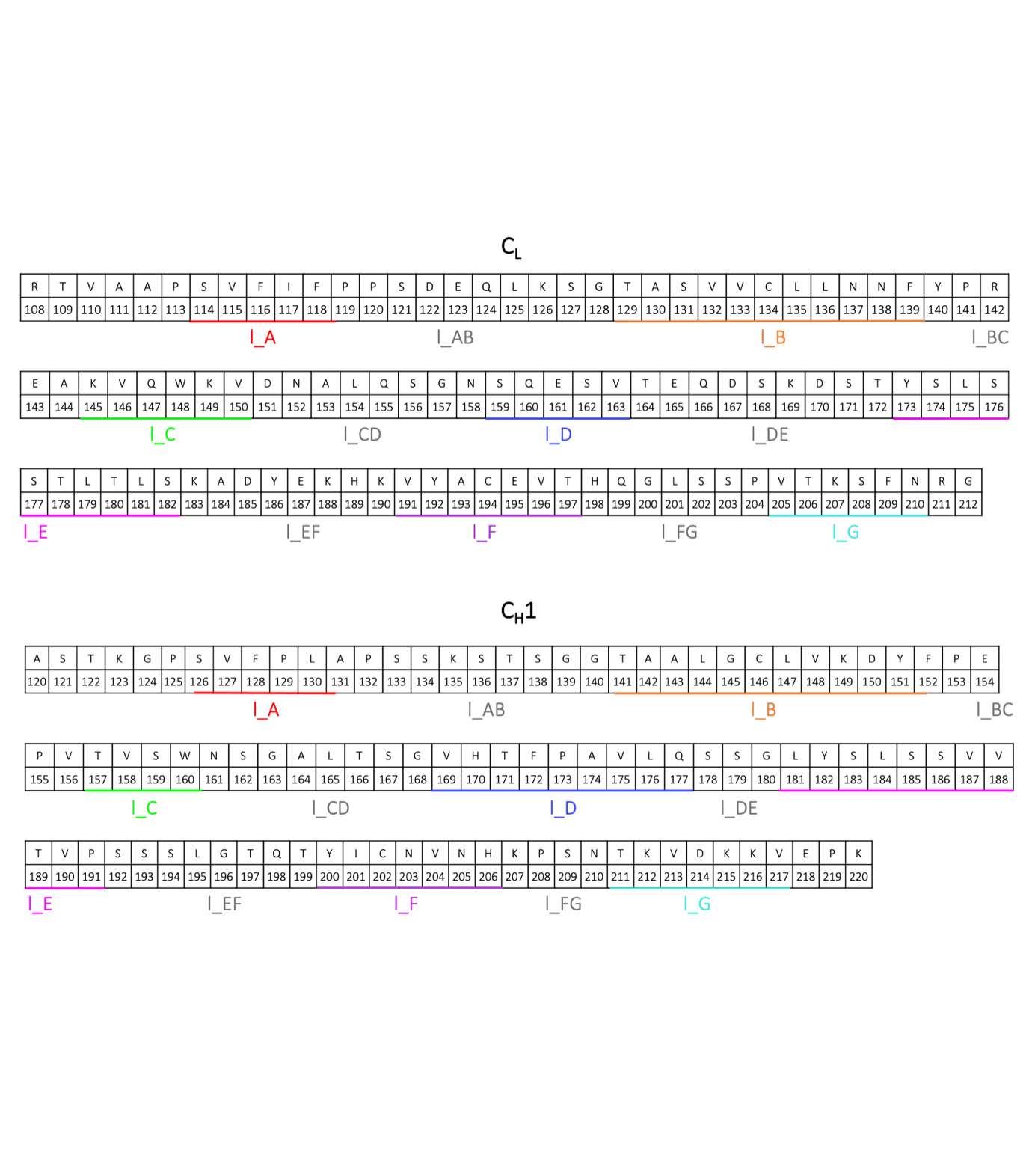
**SI Fig.8. C_H_1-C_L_ numeration.** The picture represents the numeration in the C_H_1-C_L_ region. The different β-strands are highlighted and colored according to the coloring scheme presented in **SI Fig.4.**

**
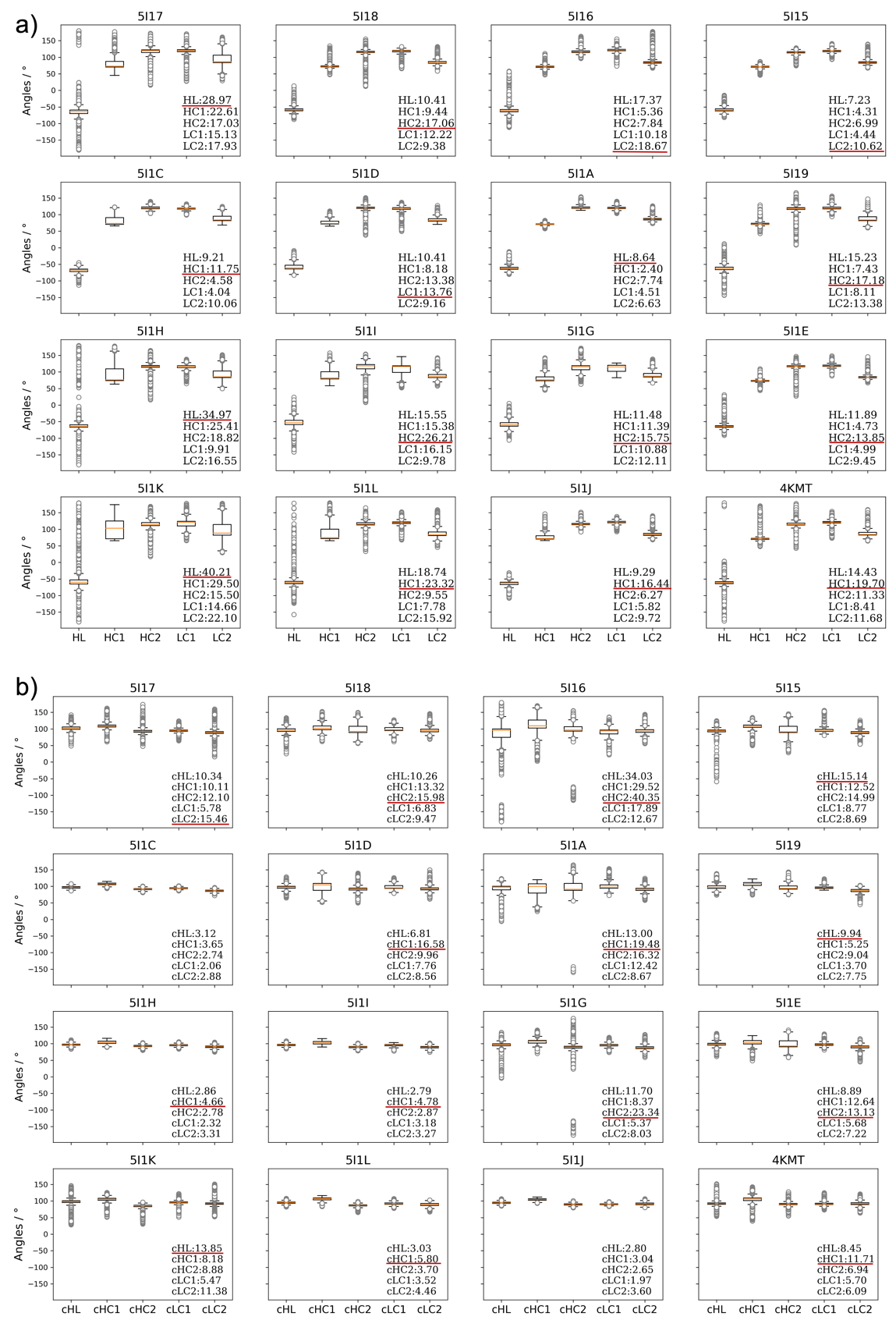
SI Fig.9. Shift in the interdomain orientation during dissociation. a)** Box plots showing the shifts in the V_H_-V_L_ angles for each system in the bound-encounter states. The standard deviation of each angle is also provided and the angle showing the highest deviation is underlined. **b)** Box plots showing the shifts in the C_H_1-C_L_ angles for each system in the bound-encounter states. The standard deviation of each angle is also provided and the angle showing the highest deviation is underlined.
